# Prevalence and factors associated with diarrheal diseases among children below five years in selected slum settlements in Entebbe municipality, Wakiso district, Uganda

**DOI:** 10.1186/s12887-022-03448-2

**Published:** 2022-07-07

**Authors:** Robinah Nantege, Dickson Kajoba, Christopher Ddamulira, Fred Ndoboli, David Ndungutse

**Affiliations:** 1 Department of Public Health, School of Graduate Studies, Bugema University, Kampala, Uganda; 2 Ministry of Water and Environment, Kampala, Uganda; 3 Department of Paediatrics and Child Health, Faculty of Clinical Medicine and Dentistry, Kampala International University, Kampala, Uganda

**Keywords:** Diarrhea, Children under 5 years, Risk factors, Slum settlements, Entebbe

## Abstract

**Background:**

Despite global interventions to prevent and control diarrhea, it still remains a public health problem leading to childhood morbidity and mortality majorly in developing countries. In Uganda, diarrhea is amongst the five leading causes of under-five mortality, contributing to more than 140,000 deaths every year and this accounts for 7.1% of all under-five mortalities in Uganda. Efforts to prevent and lower diarrheal diseases need to be informed by data on determinants of diarrhea. The study assessed factors associated with diarrheal diseases among children below five years in selected slum settlements in Entebbe municipality, Wakiso District, Uganda.

**Methods:**

The study employed a cross-sectional study design covering 384 randomly selected households having children < 5 years old in the study area using quantitative research methods. Data was collected using close-ended questionnaires and diarrhea disease history was captured for the last month before the survey. Bivariate and multivariate logistic regression analyses were used to identify the risk factors associated with childhood diarrhea considering a 95% confidence level.

**Results:**

The prevalence of Diarrhea disease in children among the selected slum settlements in Entebbe municipality was found to be at 62.4%. Access to water from a protected water source (deep well and borehole), presence of a vent in toilets, age, and child birth weight were found to be significantly associated with diarrheal diseases among children below five years in the selected slum settlements in Entebbe municipality.

**Conclusion:**

The prevalence of childhood diarrhea among children < 5 years of age in selected slums of Entebbe municipality was found high. Use of water from a protected source, presence of a vent in toilets, age, child birth and weight were identified as predictors of diarrhea occurrence. These findings imply that community health education is urgently needed for fighting childhood diarrhea in the study area to eliminate the predisposing factors to diarrhea.

**Supplementary Information:**

The online version contains supplementary material available at 10.1186/s12887-022-03448-2.

## Background

Diarrhea remains a leading cause of mortality and morbidity despite global efforts like provision of water, promotion of breastfeeding, and proper hygiene to control its prevalence. It accounts for 3.6% of the global burden of disease [1] and one of leading killer diseases in children, with 8% mortality among all deaths in children below 5 years globally [2] and 21% in developing countries [3]. The number of deaths due to diarrhea is even higher than death due to diseases like AIDS, malaria, and measles in the study groups. Every year, 760,000 children die from diarrhea, which is equivalent to 2195 children dying every day or losing nearly 32 school buses full of children every day [4].

In Uganda, diarrhea remains among the five leading causes of under-five mortality, accounting for 8% of the 85,000 under five children mortality [3], and a national prevalence of 20% among children under 5 years [5]. However, Northern Uganda reports diarrheal prevalence ranging from 29.1% to 41.3% [6]. In Uganda, the most prevalent etiological organisms in acute diarrhea include Bacterial or protozoa (43.91%), followed by parasites (32.43%), and viral (2.02%) [7]. Among the bacterial enteric pathogens *Escherichia coli* and *Shigella* species are the most isolated [8].

According to UDHS report [9], 17% of the total population in Entebbe municipality is children below 5 years. 1490 homesteads in the municipality use unprotected water sources while 11,535 households use non improved toilet facilities for human waste disposal [10] risking children below 5 years to diarrheal diseases.

## Methods

### Study area

Geographically, the study was carried out in Busambaga and Kitubulu slum settlement villages in Entebbe municipality in Wakiso district in Uganda. Entebbe municipality lies at 0^o.^04^o^N, 32^0.^28^0^E on the peninsula of Lake Victoria, approximately 37 km southwest of Kampala, Uganda’s capital, covering a total area of 56.2 km^2^, out of which 20km^2^ is water(www.mirror.unhabitat.org).

Entebbe municipality forms part of the 16 administrative units of Wakiso District with a total of 10,217 children under 5 years and majority of the people are small scale subsistence farmers [10]. Additionally, the municipality has 1490 homesteads with unprotected water sources while 11,535 households utilize non improved toilet facilities for human waste disposal [10]. Kitubulu and Busambaga are some of the informal resettlement areas in the municipality and they are reported to have the highest burden of children under 5 years diagnosed with diarrheas in the municipality (Entebbe Hospital HMIS, 2017).

### Research design and methodology

The study was conducted using a cross-sectional study design employing quantitative study approaches in two slum settlements purposively selected out of 3 slum settlements in Entebbe municipality. Entebbe municipality where Kitubulu and Busambaga settlements are found, has a total of 10,217 number of children under 5 years [10]. Using the sampling framework provided by Cochran (Cochran, 1977), a sample size of 384 respondents was considered for the study but samples were drawn from each cluster for inclusion into the study. Using clustering techniques, the population was clustered into settlements, that is; Kitubulu and Busambaga settlement, from which households were selected using convenience sampling based on the inclusion criteria. Only children below 5 years from households where a mother or caretaker was present were considered in the study and in households where more than one child below 5 years existed, a lottery method was used to select one. Trained research assistants administered the questionnaires and the dependent variable was the occurrence of diarrhea among children below 5 years within a month before the study. The case definition for an episode of diarrhoea was 3 or more episodes of diarrhoea in a 24 h period.

### Data analysis

Descriptive analysis using frequency and percentages were used to summarize the independent and dependent variables using SPSS version 22. Multivariant logistic regression was used to obtain the associations between diarrhea among children below 5 years and associated factors and the adjusted odds ratios [AORs] of diarrhea with 95% confidence interval [CIs] and *P*-value < 0.05 were used to describe associations. First, we conducted bivariate analyses to determine the associations between diarrhea and other associated factors using chi-square and binary logistic regression. Only significant variables with p values less than 0.05 in bivariate analyses were included in the final multivariable logistic regression.

## Results

### Socio-Demographic and hygiene characteristics of the participants

 A total of 378 mother/ Guardian pairs were enrolled into the study with a 100% respondence rate. Majority of the children were aged between 1–2. years with 334 (88.4%) exclusively breastfed for 6 months and 237 (62.7) had received their 2 doses of Rota virus vaccination. 26–30 years was the modal age of the mothers in the study with 60.3% having formal education and consequently 224 (59.2%) having some form of employment whether home based or away from home. Furthermore, 91.5% of the homesteads visited had permanent residential homes with large family sizes (67.5%) and poor general household hygiene (59%). Despite the large family sizes, 56.1% of the households had 1–2 children and domestic water was from a treated source (62.7%) and mothers practiced improper handwashing behaviors (83.9%) as shown in Table 1.

**Table 1:** Socio-demographic and hygienic characteristics of the study participants

Category	Frequency	percentage
**Demographic factors**		
**Maternal Age (years)**		
Less than 20	77	20.4
20–25	47	12.4
26–30	121	32.0
30–35	65	17.2
Above 35	68	18.0
**Level of education**		
Non-formal	150	39.7
Formal	228	60.3
**Employment**		
House wife	151	39.9
Home based employment	182	48.1
Working away from home	42	11.1
**Mother/guardians Income (Ugandan shillings)**		
None	151	39.9
50,000- 150,000	71	18.8
150,000–250,000	114	30.2
250,000- 350,000	29	7.7
Above 350,000	13	3.4
**Residential house**		
Temporary	32	8.5
Permanent	346	91.5
**Family size**		
Small	123	32.5
Large	255	67.5
**Number of children**		
1 to 2	212	56.1
3 to 4	108	28.6
5 and above	58	15.3
**General homestead sanitation**		
Good	155	41.0
Poor	223	59.0
**Sources of water for domestic use**		
Open source	44	11.6
Protected	97	25.7
Treated water	237	62.7
**Maternal Hand washing behavior**		
Improper	317	83.9
Proper	61	16.1
**Child characteristics**		
**Age (years)**		
Less than 1	10	2.6
1.0 -2.9	197	52.1
3 and above	171	45.2
**Birth weight (kgs)**		
Low birth weight	25	6.6
Normal weight	234	61.9
Big baby	119	31.5
**Cessation of Breastfeeding**		
Early weaning	230	60.8
Not early weaning	148	39.1
**Introduction of mixed feeds**		
Less than 6 months	44	11.6
6 months and above	334	88.4
**Rotavirus immunization**		
Not immunized	26	6.9
Partial	115	30.4
Complete	237	62.7

### Prevalence of diarrheal diseases

**Fig. 1:**
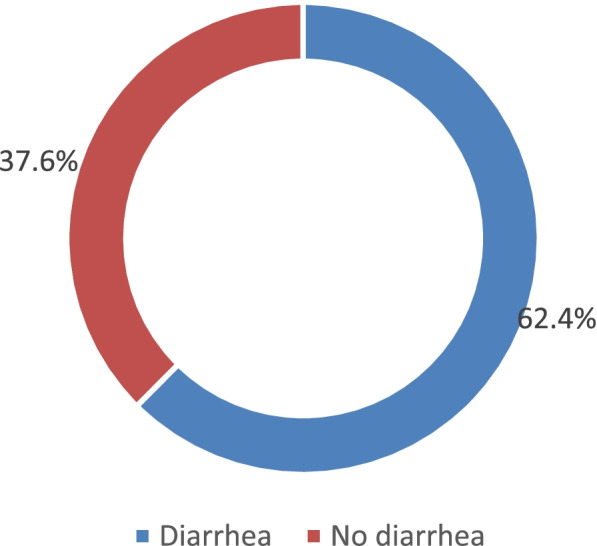
Figure showing prevalence of diarrhea among study participants

From the 378 children below 5 years that were surveyed, the study indicates that majority 236(62.4%) of the children below 5 years in the village slum settlements of Entebbe municipality had suffered from diarrhea the past 1 month before the study was carried out and only 142(37.6%) had not suffered from diarrhea the previous month as shown in Fig.1 below.

### Factors associated with diarrhea disease

At bivariant level analysis, the following were found significantly associated with the occurrence of diarrhea among children below 5 years in the study area: Among social demographic factors, Family size (X^2^ = 7.147, df = 1, *p*-value = 0.008) and number of children in a homestead (X^2^ = 8.534, df = 2, *P*-value = 0.014) were found significant. Among environment-related factors, source of water (χ2 = 26.318, df = 2, *p* < 0.001) and latrine related factors, the presence of latrine (χ2 = 6.356, df = 2, *p* < 0.001) and latrine cleanliness (χ2 = 24.026, df = 2, *p* < 0.001) were significantly responsible for diarrhea.

Furthermore, behavioral factors like using separate feeding utensils for the baby (χ2 = 29.666, df = 1, *P* < 0.001), boiling of feeding utensils in water (χ2 = 28.806, df = 1, *P* < 0.001) and hand washing behavior (χ2 = 13.903 df = 1, *P* < 0.001) were significantly associated. Additionally, child factors like age (χ^2^ = 15.204, df = 2, *P* < 0.001), Birth weight (χ^2^ = 35.288, df = 2, *P* < 0.001) cessation of breastfeeding (χ^2^ = 24.178, df = 1, *P* < 0.001) and rotavirus (χ^2^ = 23.868, df = 2, *P* < 0.001) were also significantly associated with diarrhea.

Factors that were significant at bivariant analysis were subjected further to multivariant analysis using forward selection analysis. Family size, maternal handwashing behavior, water source, child’s age, birthweight, and toilet cleanliness were statistically significant contributors to diarrhea.

Children residing in large families (AOR = 2.224 95% C.I 1.183–4.182, *p*-Value = 0.013) had a 2.224 times increased risk of suffering from diarrhea compared to the counterparts in small families whereas improper maternal handwashing (AOR = 4.645 955C.I 1.910–11.296, *p* = 0.001) contributed to 4.645 higher odds compared to those with proper handwashing behaviors.

Obtaining water from a protected water source (AOR = 0.265, 95% CI 0.108–0.650, *p* = 0.004) was associated with a 73.5% reduction in diarrhea disease when compared with unprotected water sources like lakes and shallow wells. Regarding child factors, being 3 years and above (AOR = 0.513, 95%CI 0.294–0.895, *p*-value = 0.019) was protective against diarrhea with 0.513 reduced odds when compared to those below 1 year of age. Furthermore, being born with a normal birthweight (2.5–3.9kgs) (AOR = 0.125, 95% CI 0.034–0.456, *P* = 0.002) was also associated with 87.5% reduced diarrhea chances when compared to low-birth-weight children. The study as well found that having a vent piped toilet (AOR = 0.503, 95% C.I 0.281–0.900, *P*-value = 0.021) was protective against diarrhea among children below 5 years with 0.503 times of odds reduction when compared to toilet having lid covers in slum settlements in Entebbe municipality. This is as shown in Tables 2 and 3

**Table 2:** A table showing the results of bivariant logistic analysis of factors associated with diarrhoea among children below 5 years in slum settlements in Entebbe municipality, Uganda

	**Frequency**				
**Category**	**No diarrhoea *N* (%)**	**Diarrhoea** ***N*** **(%)**	**X**^**2**^	**Df**	***P*-value**
**Demographic factors** **Age (years)**					
Less than 20	24(16.9)	53(22.5)			
20–25	23(16.2)	24(10.2)			
26–30	53(37.3)	68(28.8)	8.792	4	0.067
30–35	18(12.7)	47(19.9)			
Above 35	24(16.9)	44(18.6)			
**Level of education**					
Non-formal	49(34.5)	101(42.8)	2.545	1	0.111
Formal	93(65.5)	135(57.2)			
**Employment**					
House wife	61(49.2)	90(43.3)			
Home based employment	45(31.7)	94(45.2)	2.639	2	0.267
Working away from home	18(19.1)	24(11.6)			
**Mother/guardians Income (Ugandan shillings)**					
None	58(40.8)	93(39.4)			
50,000- 150,000	22(15.5)	49(20.8)			
150,000–250,000	47(33.1)	67(28.4)	5.388	4	0.250
250,000- 350,000	13(9.2)	16(6.8)			
Above 350,000	2(1.4)	11(4.7)			
**Residential house**					
Temporary	17(12.0)	15(6.4)	3.608	1	0.057
Permanent	125(88.0)	221(93.6)			
**Family size**					
Small	58(40.8)	65(27.5)	7.147	1	0.008*
Large	84(59.2)	171(72.5)			
**Number of children**					
1 to 2	91(64.1)	121(51.3)			
3 to 4	38(26.8)	70(29.7)	8.534	2	0.014*
5 and above	13(9.2)	45(19.1)			
**Homestead sanitation factors**					
General homestead sanitation					
Good	63 (44.4)	92(39.0)	1.062	1	0.303
Poor	79(55.6)	144 (61.0)			
**Water related factors**					
**Sources of water for domestic use**					
Open source	26(18.3)	18(7.6)			
Protected	50(35.2)	47(19.6)	26.318	2	0.000*
Treated water	66(46.5)	171(72.5)			
**Latrine related factors**					
Presence of toilet/latrine					
Yes	135(95.1)	234(99.2)	6.356	1	0.012*
No	7(4.9)	2(0.8)			
**Sharing of toilets**					
Yes	112(78.9)	184(78.0)	0.043	1	0.836
No	30(21.1)	52(22.0)			
**Toilet type**					
Local latrine	125(88.0)	212(89.8)	0.298	1	0.585
VIP toilet	17(12.0)	24(10.2)			
**Cleanliness**					
Lid cover	79(55.6)	72(30.5)			
Has VIP and clean	11(7.7)	21(8.9)	24.026	2	0.000*
Houseflies	52(36.6)	143(60.6)			
**Number of people sharing**					
None	28(19.7)	49(20.8)			
1–3	43(30.3)	87(36.9)	4.678	3	0.197
4–6	41(28.9)	46(19.5)			
Above 6	30(21.1)	54(22.9)			
**Feeding related factors** **Use separate feeding utensils for the baby**					
No	79(55.6)	65(27.5)	29.666	1	0.000*
Yes	63(44.4)	171(72.5)			
**Boil of feeding equipment**					
No	72(50.7)	56(23.7)	28.806	1	0.000*
Yes	70(49.3)	180(76.3)			
**Hand washing behaviour**					
Improper	185(78.4)	132(93.0)	13.903	1	0.000 *
Proper	51(21.6)	10(7.0)			
**Child factors**					
**Age (years)**					
Less than 1	5(5.5)	5(2.1)			
1.0 -2.9	91(64.1)	106(44.9)	15.204	2	0.000*
3 and above	46(32.4)	125(53.0)			
**Birth weight (kgs)**					
Low birth weight	19(13.4)	6(2.5)			
Normal weight	100(70.4)	134(56.8)	35.288	2	0.000*
Big baby	23(16.2)	96(40.7)			
**Cessation of Breastfeeding**					
Early weaning	109(76.8)	121(51.3)	24.178	1	0.000*
Not early weaning	33(23.2)	115(48.7)			
**Introduction of mixed feeds**					
Less than 6 months	18(12.7)	26(11.0)	0.237	1	0.626
6 months and above	124(87.3)	210(89.0)			
**Rotavirus immunization**					
Not immunised	4 (2.8)	22(9.3)			
Partial	27(19.0)	88(37.3)	23.868	2	0.000*
Complete	111(78.2)	126(53.4)			

Where: * *p*- value less than 0.05

**Table 3:** A table showing Multivariant Logistic Regression of Factors Associated with Diarrhea among Children

	**Diarrhoea status**			
	**Yes** ***N*(%)**	**No** ***N*(%)**	**COR (95%CI)**	**AOR (95%CI)**
**Social Demographic factors**				
Family size				
Small	65(27.5)	58(40.8	-1-	-1-
Large	171(72.5)	84(59.2)	1.816(1.170–2.820)	2.224(1.183–4.182) *
**Environmental factors**				
Hand washing behaviour				
Proper	51(21.6)	10(7.0)	-1-	-1-
Improper	185(78.4)	132(93.0)	3.639(1.782–7.429)	4.645(1.910–11.296) *
**Water source**				
Open source	26(18.3)	18(7.6)	-1-	-1-
Protected water source	50(35.2)	47(19.6)	0.267(0.137–0.519)	0.265(0.108–0.650) *
Treated water	66(46.5)	171(72.5)	0.363(0.222–0.592)	1.034(0.505–2.117)
**Age of the child**				
Less than 1 year	5(2.1)	5(5.5)	-1-	-1-
1.0 to 2.9 years	27(11.4)	38(26.8)	0.368(0.102–1.330)	0.290(0.047–1.794)
3 years and above	79(33.5)	53(37.3)	0.261(0.144–0.475)	0.513(0.294–0.895) *
**Birth weight**				
Low birth weight	6(2.5)	19(13.4)	-1-	-1-
Normal weight	134(56.8)	100(70.4)	0.0.076(0.027–0.211)	0.125(0.034–0.456) *
Big baby	96(40.7)	23(16.2)	0.321(0.190–0.542)	0.508(0.262–0.986)
**Cleanliness of toilet**				
Has cover lid	72(30.5)	79(55.6)	-1-	-1-
Has VIP	21(8.9)	11(7.7)	0.311(0.211–0.520)	0.503(0.281–0.900) *
Houseflies	143(60.6)	52(36.6)	0.694(0.313–1.538)	0.990(0.303–2.973)

Key * *P*- value less than 0.05

## Discussion

The study assessed the prevalence and factors associated with diarrheal diseases among children below 5 years in selected slum settlements in Entebbe municipality, Wakiso district, Uganda. There was an overall period prevalence of 62.3% of diarrheal diseases in the month preceding the study which shows a very high diarrheal burden among the children in slum settlements. This could be attributable to the lack of basic amenities for proper health and considerable independence where they play unsupervised within the community environment which is highly prone to high level of contamination [11].

The findings are consistent with the 2017 WHO report that 50% of the 2 million deaths worldwide are due to watery diarrhea, 15% persistent diarrhea, and 35% due to dysentery (WHO, 2017). The findings are way higher than that observed in Senegal 26% (range: 7.1–43.6) [11], South Africa 15.3% (range: 8.6–24.2%) [12] and Nepal 40.2% [13]. The observed difference could be because of the study duration where other studies used 2 weeks compared to the one month used in this current study.

The study showed a significant relationship with family size, maternal handwashing behavior, source of water, child age, birthweight, and toilet type. The study showed a 2.224 increased risk for diarrhea among children residing in large families (AOR = 2.224 95% C.I 1.183–4.182, *p* = 0.013) when compared to those in small families. This is because there is a higher likelihood of infective diarrhea spread from agents being easily transmitted from one person to another, especially in large families increasing their risk [14]. The study finding agree with a study in Ethiopia where there was 91.2% less likelihood of diarrhea prevention in large families [15] and 22.4 increased risk with families having more than 3 children under 5 years [14].

The study showed 4.645 increased odds of suffering from diarrhea in a child whose mother had poor handwashing behavior compared to those with proper handwashing behavior. Dirty hands serve as portals for carrying infectious pathogens to the skin of the child, especially the hands and further inoculation into the mouth, thus increasing diarrhea [16]. That further emphasized the fact that the intervention of hand washing with soap and water, together with sanitation and hygiene (WASH) educational intervention reduced the diarrhoea incidence by 35% among children below 5 years in eastern Ethiopia [17]. Obtaining water from a protected source reduced the odds of diarrheal disease by 73.5% when compared with an open water source. Open water sources are highly prone to contamination especially from fecal flow and sharing with animals [18] the findings agree with a study in Nigeria where there was a high prevalence of diarrhea in children with un improved water sources (11.2% vs 9.5%) and an increasing risk by 1.20 (95% C.I 1.11–1.30) [19].

Child’s age especially above 3 years, was associated with 48.7% reduced chances of having diarrhea when compared with those less than 1 year (AOR = 0.513, 95%CI 0.294–0.895, *p* = 0.019). This is due to the fact that children below 1 year have low immunity, haven’t received measles vaccination and introduction of complementary feeds and trying out new feeds which usually coincides with diarrhea in developing countries [20]. Such a finding agrees with a study by Pintu (2020) [21] and Vasconcelos et al. (2018) [22] that there’s a higher risk in the age group 0–11 months and that diarrhea reduces with an increase in age reducing diarrhea by 43–70% by age above 24 months.

Birthweight was significantly associated with diarrhea among children below 5 years with normal weight reducing diarrhea by 87.5% (AOR = 0.125, 95% CI 0.034–0.456, *P* = 0.002) in comparison to those with low birth weight. The result is attributable to the fact that low birth weight (< 2.5kgs) is a key determinant for infectious diseases including diarrhea due to low immunity. Additionally, low birth weight is associated with undernutrition (stunting 57%, underweight -15%, and wasting 51%), which is a great predictor of diarrheal diseases [23]. The study findings agree with a study by Kumer and Bokar (2018) who found a 1.38 times higher risk of diarrhea among low birth weight babies while Singh and Singh (2014) [24] found a 51% reduction in diarrhea among normal weight babies compared to low birthweight babies.

Having a vent pipe on the toilet as a cleanliness factor was also associated with 0.503 reduced odds for diarrhea (AOR = 0.503, 95% C.I 0.281–0.900, *P* = 0.021) compared to having lid cover toilets. Presence of a vent pipe helps to reduce bad smell and houseflies in the toilet, which breaks the diarrhea transmission pathway and reduces the spread of diarrhea pathogens by houseflies [25] furthermore having an improved latrine plays a role in reducing diarrhea cases and mortality to up-to 30% in children [26].

## Conclusion

The study shows a huge burden of diarrhea among children below 5 years in slum settlement and factors at play in the causation of diarrhea. The study findings provide a basis for strengthening strategies for diarrheal prevention in slum settlements like access to protected water sources, use of vent piped latrines, and fecal waste management. Furthermore, adherence to the focused antenatal care promotion to prevent low birthweight and premature deliveries. Diarrhea, especially in children below 5 years, is associated with high morbidity and mortality, thus health stakeholders and supporting bodies need to engage more on how to reduce the burden in slum areas.

## Supplementary Information

**Additional file 1. **

## Data Availability

Data set generated and used for analysis in this current study has been submitted as supplementary file.
